# The IBRA (implant breast reconstruction evaluation) study: a prospective multicentre cohort study to inform the feasibility, design and conduct of a pragmatic randomised clinical trial comparing new approaches to implant-based breast reconstruction

**DOI:** 10.1186/1745-6215-16-S2-P12

**Published:** 2015-11-16

**Authors:** Shelley Potter, Jane Blazeby

**Affiliations:** 1 Breast Reconstruction Research Collaborative, Nationwide

## Introduction

Implant-based breast reconstruction (IBBR) is the most commonly-performed reconstructive procedure in the UK. The introduction of techniques to augment the subpectoral pocket has revolutionalised the procedure, but there is a lack of high-quality outcome data to support the safety or efficacy of these techniques. Randomised controlled trials (RCTs) are the best way of comparing treatments but surgical RCTs are challenging. The iBRA (implant Breast Reconstruction evAluation) study aims to determine the feasibility of conducting a pragmatic-RCT to examine the effectiveness of new approaches to IBBR.

## Methods

The iBRA study is a trainee-led research collaborative project with 4-phases:

**Phase 1**–A national practice questionnaire (NPQ).

**Phase 2**–A prospective cohort-study of consecutive patients undergoing IBBR to evaluate the clinical and patient-reported-outcomes of surgery.

**Phase 3**–An IBBR-RCT acceptability-survey to explore patients’ and surgeons’ views of proposed trial designs.

**Phase 4-**Design of the definitive RCT.

iBRA will document current-practice, explore potential comparators, identify relevant outcome measures, inform selection-criteria. trial conduct and sample-size to determine whether a future trial is feasible.

## Results

Since May-2014, 90-units have agreed to participate and 56 have contributed to the NPQ. 653-patients have been recruited from 51-centres and the study is six-months ahead-of-schedule.

## Discussion

The iBRA study will not only determine trial-feasibility, but will also generate the best current evidence regarding the outcomes of new approaches to IBBR. Furthermore, the novel trainee collaborative methodology will build capacity by creating an infrastructure of research-active breast and plastic surgeons which will facilitate future high-quality research and ultimately improve outcomes for all women seeking reconstructive surgery.

